# Plasmonic Resonant Nanoantennas Induce Changes in the Shape and the Intensity of Infrared Spectra of Phospholipids

**DOI:** 10.3390/molecules27010062

**Published:** 2021-12-23

**Authors:** Fatima Omeis, Zahia Boubegtiten-Fezoua, Ana Filipa Santos Seica, Romain Bernard, Muhammad Haseeb Iqbal, Nicolas Javahiraly, Robrecht M. A. Vergauwe, Hicham Majjad, Fouzia Boulmedais, David Moss, Petra Hellwig

**Affiliations:** 1 Laboratoire de Bioélectrochimie et Spectroscopie, UMR 7140, CMC, Université de Strasbourg CNRS, 4 Rue Blaise Pascal, 67081 Strasbourg, France; fatima.omeis@institutoptique.fr (F.O.); boubegtiten@unistra.fr (Z.B.-F.); santosseica@unistra.fr (A.F.S.S.); n.javahiraly@unistra.fr (N.J.); 2 University of Strasbourg Institute for Advanced Studies (USIAS), 4 Rue Blaise Pascal, 67081 Strasbourg, France; 3 Institut de Physique et Chimie des Matériaux de Strasbourg, Université de Strasbourg CNRS, UMR 7504, 23 Rue du Loess, BP 43, CEDEX 2, 67034 Strasbourg, France; romain.bernard@ipcms.unistra.fr (R.B.); hicham.majjad@ipcms.unistra.fr (H.M.); 4 Institut Charles Sadron, University of Strasbourg CNRS, UPR 22, 67034 Strasbourg, France; hasee.iqbal@etu.unistra.fr (M.H.I.); fouzia.boulmedais@ics-cnrs.unistra.fr (F.B.); 5 NanoScience Center, Survontie 9 C, 40500 Jyväskylä, Finland; robrecht.ma.vergauwe@jyu.fi; 6 Institute for Beam Physics and Technology, Karlsruhe Institute of Technology, Hermann-von-Helmholtz-Platz 1, 76344 Eggenstein-Leopoldshafen, Germany; david.moss@kit.edu

**Keywords:** nanoantennas, DPPTE, bilayers, SEIRAS, enhancement, AFM

## Abstract

Surface enhanced infrared absorption spectroscopic studies (SEIRAS) as a technique to study biological molecules in extremely low concentrations is greatly evolving. In order to use the technique for identification of the structure and interactions of such biological molecules, it is necessary to identify the effects of the plasmonic electric-field enhancement on the spectral signature. In this study the spectral properties of 1,2-Dipalmitoyl-sn-glycero-3 phosphothioethanol (DPPTE) phospholipid immobilized on gold nanoantennas, specifically designed to enhance the vibrational fingerprints of lipid molecules were studied. An AFM study demonstrates an organization of the DPPTE phospholipid in bilayers on the nanoantenna structure. The spectral data were compared to SEIRAS active gold surfaces based on nanoparticles, plain gold and plain substrate (Si) for different temperatures. The shape of the infrared signals, the peak positions and their relative intensities were found to be sensitive to the type of surface and the presence of an enhancement. The strongest shifts in position and intensity were seen for the nanoantennas, and a smaller effect was seen for the DPPTE immobilized on gold nanoparticles. This information is crucial for interpretation of data obtained for biological molecules measured on such structures, for future application in nanodevices for biologically or medically relevant samples.

## 1. Introduction

Plasmonics have become one of the most vibrant areas in research with technological innovations impacting fields from telecommunications to medicine. Gold nanoantennas control infrared and visible light at the nanoscale through excitation of collective electron oscillations known as plasmons (or localized surface plasmons), concentrating into small subdiffraction-limited “hot spots” in the feed-gap of the antenna.

Huge local field intensities of several orders of magnitude higher than the incident one have been achieved within these structures, allowing the study of light-matter interaction at the nanoscale and to detect picomolar amounts of biological samples, including large protein complexes. An important achievement is that the optical resonances can be tuned with the geometrical shape and dielectric constant of the nanostructures and with the illumination properties (polarization, wavelength) and can thus be used for sensitive infrared detection.

In several studies, simple linear nanorods with micrometer lengths and nanometer cross-sectional geometries have been used [1]. The plasmonic response critically depended on both, the rod length and radius. When the vibrational frequency of the molecules in the vicinity of the hot spots matches the plasmonic resonant frequency, a coupling between the two modes occurs resulting in an increase in the amplitude of the molecular vibrational signal [2,3,4,5,6,7,8]. Theoretical studies predict an influence of the resonance of the nanostructure on the spectral properties of the studied molecules [9].

First studies on the formation of the biomimetic membranes [10,11] and on the interaction of lipid membrane with peptides on nanoantennas have been reported [10]. The lateral organization of lipid model membranes was described with microsecond time resolution using in-plane plasmonic nanogap antennas [12]. In addition, an infrared plasmonic biosensor was described that allows the detection of supported lipid bilayers and their real-time formation kinetics in an aqueous solution [10]. The lipid bilayer formation on this plasmonic surface was monitored by considering the time evolution of the absorbance signal of specific vibrational bands of the lipid, CH_2_. To best of our knowledge no literature data concerning the modification of the infrared signature of phospholipids integrated with nanoantennas were reported. However, organic model molecules were used to investigate the behavior of the line-shape of the vibrational signals of molecular layers adsorbed on Au nanoantenna, such as octadecanthiol [13], poly(methyl methacrylate) [14], 4,4′-bis(*N*-carbazolyl)-1,1′-biphenyl [15,16]. The vibrational signals of the molecules exhibit asymmetric Fano profile like shapes and spectral shifts in the absorption peaks, resulting from the strong interaction between the plasmonic resonances of nanoantennas and the molecular dipoles of the molecules.

**Figure 1:**
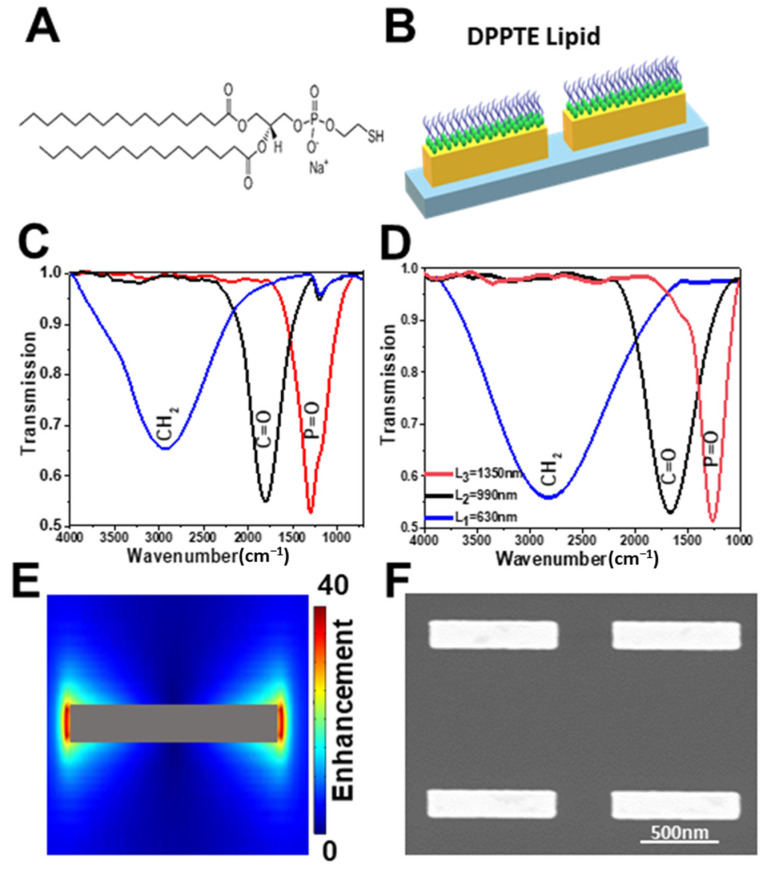
(**A**) Structure of the DPPTE (1,2-dipalmitoyl-sn-glycero-3-phosphothioethanol, sodium salt). (**B**) Schematic representation of the DPPTE thiolipid attached to the nanoantennas. (**C**) Measured relative transmittance of gold nanoantenna arrays of different lengths (L1 = 630 nm, L2 = 990 nm, L3 = 1350 nm) while the height and the width are h = 100 nm and w = 150 nm. These different lengths of the nanoantennas were used to target the spectral regions associated with CH_2_ symmetric and antisymmetric stretching, C=O stretching, P=O antisymmetric stretching modes of the lipid as indicated by blue, black and red lines, respectively. (**D**) Simulated relative transmittance spectra of gold nanoantennas. (**E**) Electric field (E/E0) enhancement. The color scale (shading) indicates the magnitude of the enhancement. (**F**) Scanning electron microscopy (SEM) image of a selected antenna array with a length of 900 nm fabricated on a Si substrate.

In the present work, we provide new insight into the infrared spectroscopic signatures of phospholipids integrated with gold nanoantennas which is crucial information for understanding the reactivity of the membranes. We immobilized the phospholipid molecules of 1,2-Dipalmitoyl-sn-glycero-3 phosphothioethanol (DPPTE) onto plasmonic nanoantennas (Figure 1A,B). The geometry of individual nanoantennas was selected to match the molecular vibrational modes [17,18,19] of the characteristic functional groups of the phospholipid, namely the ν(CH_2_), ν(C=O), and the ν(P=O) bands (Figure 1C,D). The data were directly compared to the same lipid on plain gold and Au nanoparticles (AuNPs) surfaces.

## 2. Results and Discussion

### 2.1. Characterization of the Gold Nanoantennas

Figure 1C,D show the experimental and the simulated infrared measurements of the relative transmission, for the three lengths of the nanoantennas (L1 = 630 nm, L2 = 990 nm and L3 = 1350 nm) used in the study. The geometrical parameters of the nanoantennas were tailored to excite the plasmonic resonance around 2814 cm^−1^, 1662 cm^−1^ and 1258 cm^−1^, which correspond to the enhancement of the electric field (EF) in the vibrational bands regions associated with the stretching modes of the CH_2_, C=O, and PO_2_^−^, that correspond to the most important functional groups of the lipids.

As mentioned above, it was previously demonstrated that the resonance frequency of mid-IR nanoantennas using a single nanoantenna length can be spectrally tuned to overlap with the characteristic vibrational modes of a target biomolecule [10,17]. The exaltation of the electric field (EF) at a specific wavenumber is concentrated at the edges around the nanoantennas, where the vibrational signal of the targeted molecules is enhanced (Figure 1E). Figure 1F shows the representative scanning electron microscopy (SEM) image of the nanoantenna with length of 900 nm. The control by SEM was done for all the nanoantennas studied (data not shown).

The structural stability of the nanoantennas towards temperature was controlled by Atomic Force Microscopy (AFM) measurements before (at 25 °C) (Figure S1A) and after heating to a temperature of 75 °C during 44 h (Figure S1B). Only a slight change in the lateral dimension of the nanoantenna (an increase of 6% of the width) is observed. The surface roughness decreases from approximately 4.09 to 2.13 nm after heating the sample. Thus, the structure of gold nanoantenna was shown to be stable upon exposure to high temperatures. This is in line with previous studies [20], where for example for the 200 nm thick single and polycrystalline gold films, the dielectric function remains almost intact upon heating the sample from 23 to 100 °C in the wavelength range from 370 to 2000 nm.

### 2.2. AFM Characterization of the DPPTE Bilayers

**Figure 2:**
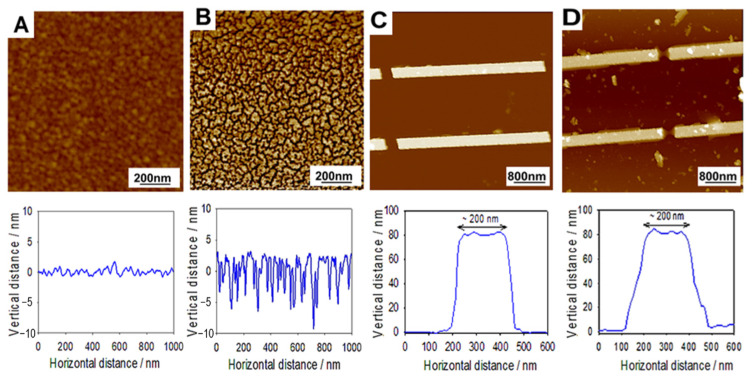
(**A**,**B**) Typical AFM height images, obtained in Peak Force Tapping mode (ScanAsyst) and in dry state, along with corresponding cross-section profiles of 7 nm Au layer (z-scale 10 nm) and (**C**,**D**) Au nanoantennas (z-scale 200 nm). (**A**,**C**) before and (**B**,**D**) after DPPTE lipid immobilization followed by the chloroform rinsing. The transversal cross-section is presented for the Au nanoantennas.

**Figure 3:**
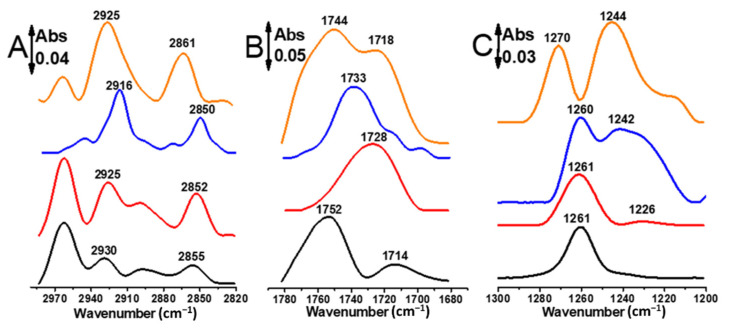
Absorbance FTIR spectra of the lipid DPPTE immobilized on silicon substrate (black lines), immobilized on gold nanoparticles (red lines), gold layer (blue lines) and on the Au nanoantennas (orange lines) measured at 25 °C. (**A**) The spectra correspond to the ν_s,as_(CH_2_), (**B**) ν(C=O) and (**C**) ν_as_ (PO_2_^−^) bands of the lipid.

**Figure 4:**
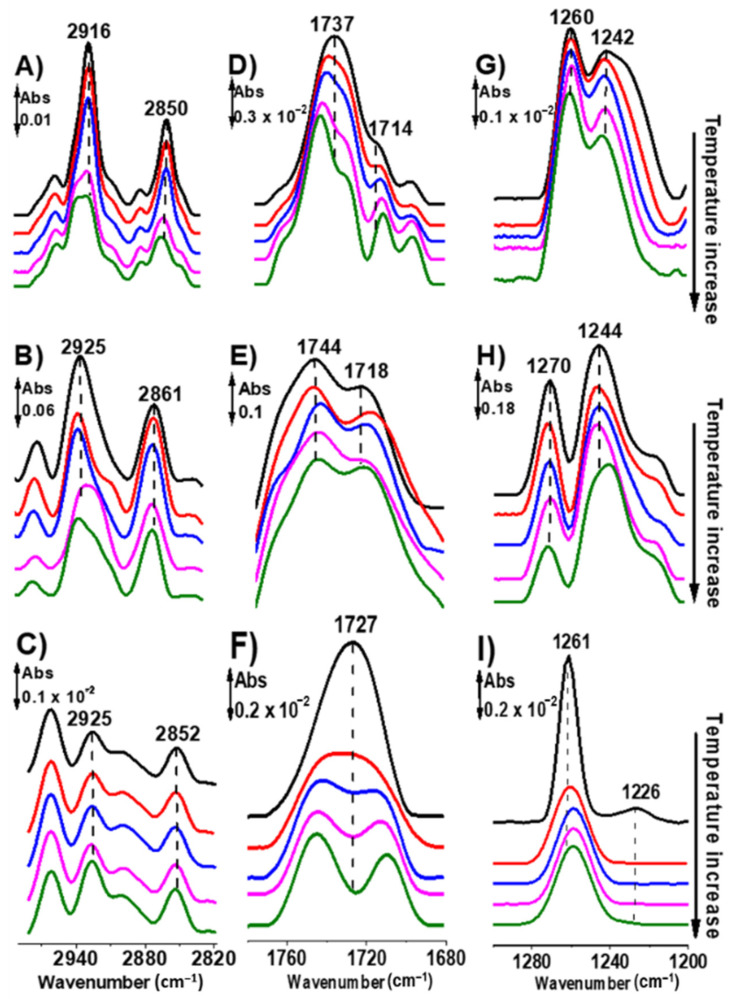
(**A**,**D**,**G**) Absorbance FTIR spectra of the temperature dependence of the lipids immobilized on a gold layer, (**B**,**E**,**H**) on the nanoantennas and (**C**,**F**,**I**) on gold nanoparticles. The spectra shown in each panel were obtained from 25 °C (top spectrum) to 75 °C (bottom spectrum): 25 °C (black line), 35 °C (red line), 45 °C (blue line), 55 °C (magenta line) and 75 °C (olive line). The panels show the spectroscopic changes occurring in the ν_s,as_(CH_2_) (**A**–**C**), the ν(C=O) (**D**–**F**) and the ν_as_(PO_2_^−^) (**G**–**I**) bands.

The immobilization of the DPPTE lipid via its thiol group on the thin film of gold and on the gold nanoantennas was controlled by AFM (Figure 2). 2D topography and cross-sectional view of these two gold substrates were performed before and after lipid immobilization followed by the rinsing step with chloroform. It is noted that the immobilization protocol of the lipid (see Materials and Methods section) used for the AFM measurements corresponds to the one used to obtain the IR data in Figure 3 and Figure 4.

The AFM analysis of 7 nm thick gold substrate confirmed significant changes in root mean square roughness (RMS) values before and after lipid immobilization followed by the chloroform rinsing step (Figure 2A,B). The RMS roughness increases from approximately 0.36 ± 0.01 to 1.5 ± 0.3 nm with the appearance of a nanotextured surface after chloroform rinsing, thus confirming the deposition of DPPTE lipid. The corresponding cross sectional profile of the nanotextured surface showed the presence of domain with an average height of 7 nm, suggesting the presence of DPPTE bilayer with 3.26 nm in lipid height in all trans configuration [21]. A previous study showed that DPPTE phospholipids incubated for 18 h in ethanol solution, formed a monolayer on the gold surface [21], whose thickness (23.5 ± 0.5 Å) is significantly lower than the lipid film obtained in this study by immersion of the gold layer substrate in 1 mM DPPTE chloroform solution for 18 h. The difference in the thickness can be due to the difference in the sample preparation. Also, it was previously shown that it is possible to modulate the properties of the DPPTE SAM by selecting the experimental incubation conditions [22,23,24].

Regarding the Au nanoantennas after lipid immobilization, followed by the rinsing step, a broader transversal cross section profile was obtained (Figure 2D and Figure S2B) in comparison to the bare nanoantennas (Figure 2C and Figure S2A). This suggests the presence of lipids on and around the nanoantennas. Despite several chloroform rinsing steps, some heterogeneous areas can be observed between Au nanoantennas on the transversal direction. This can be explained by the solvent wettability that can be altered in nano-confined areas. The cross-section of the Au nanoantennas surface showed a change in topography after the lipid immobilization (Figure S3A,B). The lipids can be observed making a bridge in between the edges of nanoantennas on the longitudinal direction (Figure 2D). The longitudinal cross-section of Au nanoantennas suggests the presence of nearly 20 nm thick lipid multilayer between the nanoantenna edges, corresponding to three DPPTE bilayers (Figure S3C,D). Due to initial high roughness of the Au nanoantennas surface, we cannot really discriminate on the formation of the lipid monolayer or multilayer on their surface.

### 2.3. Molecular Group-Specific Detection by Nanoantennas

Figure 3A–C compares the IR spectra of stretching modes of the CH_2_, C=O, and PO_2_^−^ of the lipid DPPTE immobilized on four different substrates at room temperature (25 °C), namely after deposition of the lipid on a silicon substrate of thickness (0.5 mm) (black lines), on gold nanoparticles surface that provide a good SEIRAS effect (red lines), on plain gold layer (blue lines) and on the nanoantennas (orange lines). The surfaces allow the study of the different four situations: on the plain gold surface, the lipid molecules are organized in a plain manner, whereas they are organized more randomly on the gold nanoparticles surface. In both cases we expect some small SEIRAS effect. On the silicon substrate we expected no organization and no resonance. Finally, on the Au nanoantennas the DPPTE phospholipids are organized on the rod like shape and a strong plasmonic resonance was expected.

Clear differences were seen between the data obtained on the different surfaces shown in Figure 3. In the spectral range between 3000 and 2800 cm^−1^, the CH_2_ signature of DPPTE lipid on silicon (Figure 3A, black line) and on the gold nanoparticles (Figure 3A, red line) is nearly identical. Changes in bands width, frequency and intensity occur for CH_2_ signature of the lipid on the plain gold layer (Figure 3A, blue line) and on the L1 nanoantenna (Figure 3A, orange line) when compared to the data obtained on Si and AuNPs substrates. In case of L1 nanoantenna, the bands of the lipid originating from the methylene symmetric (ν_s_(CH_2_)) and asymmetric stretching (ν_as_(CH_2_)) vibrations are found to be upshifted by 11–6 cm^−1^ and their intensities enhanced compared to the same bands of the lipid obtained on other substrates. The frequency of these bands is known to be sensitive to changes in the configuration of the acyl chains, in chain mobility and in packing [25] and here the dried DPPTE bilayer on the plain gold surface shows a high proportion of trans conformations of the acyl chain (ν_s_(CH_2_) = 2850 cm^−1^, ν_as_(CH_2_) = 2916 cm^−1^) (Figure 3A, blue line). The shifts seen for (ν_s,as_(CH_2_)) bands after adsorption of the lipid on the gold nanoantenna (Figure 3A, orange line) may partially reflect an increase in hydrocarbon chains conformational disorder. This result is compatible with the formation of DPPTE bilayers suggested by AFM measurements. On the gold nanoparticles surface (Figure 3A, red line) no specific orientation can be expected for the lipid molecules, as the Au nanoparticles create a complex structured surface. At the same time, it is not possible to exclude that the resonance affects intensity and position of the ν_s,as_(CH_2_) bands and the presence of some overlapping contributions from CH_3_ modes.

In the spectral range characteristic for the C=O vibrational mode, distinct signals can be observed by comparing the ν(C=O) bands of the lipid on all surfaces (Figure 3B). The DPPTE dried on silicon (Figure 3B, black line), gold layer (Figure 3B, blue line) and L2 gold nanoantenna (Figure 3B, orange line) substrates exhibit similar patterns of the ν(C=O) absorption (two components in the ν(C=O) band) which, interestingly, differ significantly from that exhibited by the lipid on AuNPs surface (one component in the ν(C=O) band) (Figure 3B, red line). These signals can be attributed to the stretching vibrations of the sn1-and sn2-ester carbonyl (C=O) groups of 1,2-diacyl glycerolipid [26,27,28]. Previous studies of the ν(C=O) absorption bands of diacylglycerolipids suggested that the subcomponents of the C=O band could be attributed to different subpopulations of C=O groups: a higher wavenumber component originates from C=O groups that are not hydrogen bonded and the second component corresponds to C=O groups that are involved in hydrogen bonding [29,30]. In our case, the significant changes in the contours, intensity and position of the ν(C=O) absorption band are indicative of a different structural arrangement and environment of the interfacial carbonyl groups of the thiolipid assembly on different surfaces.

In contrast to the data shown above for the ν_s,as_(CH_2_) bands, a change in the C=O bands features was observed between the thiolipid on silicon (Figure 3B, black line) and that one on AuNps substrate (Figure 3B, red line). This drastic change in the fine structure of the ν(C=O) band could mainly result from an effect of the plasmonic resonance, induced by the AuNPs, on the carbonyl stretching mode. When comparing the ν(C=O) band of the DPPTE phospholipid on AuNPs substrate (Figure 3B, red line) with its counterparts exhibited by the lipid sample on plain gold (Figure 3B, blue line) and L2 nanoantennas (Figure 3B, orange line), shifts to higher frequencies and an enhancement in the intensities are observed, mainly in the case of nanoantennas. In this case, the electric field enhancement is maximized around the L2 nanoantennas edges at 1662 cm^−1^ which may lead to enhancement of the C=O stretching mode.

The third marker band of phospholipids studied, originates from the phosphate head group. Here the adsorption of DPPTE on AuNPs surface and its disposition on silicon substrate were marked by a one strong band at 1261 cm^−1^ originating from the phosphate asymmetric stretching vibration (ν_as_(PO_2_^−^)) (Figure 3C, black and red lines) [31]. When comparing the data of the gold layer (Figure 3C, blue line) and the L3 nanoantennas (Figure 3C, orange line) with the ones obtained on the silicon and AuNPs substrates, a significant enhancement of the bands at 1242/1244 cm^−1^ is observed together with an upshift of the band from 1260 to 1270 cm^−1^. Since the ν_as_(PO_2_^−^) vibration is sensitive to hydrogen bonds formation [32,33], its shift towards higher wavenumbers is indicative of some degree of dehydration of the phosphate group in the lipid layers. The behavior of the phosphate stretching vibrational mode is more perturbed by the plasmonic resonance of the different gold nanostructures (Figure 3C, red, blue and orange lines) compared to the stretching modes of the CH_2_ and C=O groups discussed above. No simple explanation can be provided on this complex behavior of the ν_as_(PO_2_^−^) mode because of several distinct phenomena occurring during the organization and rearrangement step of the phospholipid on the gold surfaces. An overall enhancement, however, is observed for the phosphate asymmetric band on the L3 nanoantenna (Figure 3C, orange line) compared to the bands of the CH_2_ and C=O groups obtained on L1 and L2 antennas, respectively (Figure 3A,B, orange lines). This may be explained by the close vicinity of the phosphate group of the DPPTE lipid to the nanoantenna surface.

### 2.4. Temperature-Dependence of the Infrared Signal of DPPTE Bilayers on Nanoantennas

To get further insight into the interaction between the nanoantenna and the molecular vibrational modes of DPPTE, temperature dependent experiments were conducted for the thiolipid on different gold surfaces including the nanoantennas. Indeed, the temperature will influence the hydrogen bonding strength in the lipid environment but not the plasmonic resonance effects. The data are shown in Figure 4 for the three characteristic marker bands of the thiolipid discussed above: ν_s,as_(CH_2_) (Figure 4A–C), ν(C=O) (Figure 4D–F) and ν_as_(PO_2_^−^) (Figure 4 G–I).

The CH_2_ stretching bands of the lipid are sensitive to the conformation of phospholipid acyl chains [34,35] and are, therefore, often used to monitor the lipid hydrocarbon chain-melting phase transitions and, also to characterize concomitant changes in hydrocarbon chain conformational disorder [36,37,38,39].

On a plain gold surface, the ν_s_(CH_2_) and ν_as_(CH_2_) vibrations of the lipid give rise to strong bands centered near 2850 cm^−1^ and 2916 cm^−1^, respectively, at 25 °C (Figure 4A, black line). Upon increasing the temperature of the lipid on the gold layer, an upward shift in the ν_s_(CH_2_) and ν_as_(CH_2_) bands frequencies by 2 cm^−1^ and 5 cm^−1^, respectively, a significant broadening of these bands and an overall decrease in their intensities were observed (Figure 4A). These changes reflect the increase in hydrocarbon chain conformational disorder and mobility that occur with the increase of the gauche conformer content of the thiolated lipid hydrocarbon chains. Similar observations can be reported for the ester carbonyl stretching band (upshift of about 8 cm^−1^) (Figure 4D) and for the ν_as_(PO_2_^−^) band (up-shift of about 2 cm^−1^) (Figure 4G) of the lipid immobilized on a gold layer when the temperature is increased. The changes observed in the ν_s,as_(CH_2_), the ν(C=O) and the ν_as_(PO_2_^−^) bands of the thiolated lipid on plain gold layer indicate an increase in hydrocarbon chain conformational disorder, as well as a decrease in the hydration of the polar-apolar interfacial and polar head-group regions of the lipid bilayer.

When performing the same temperature dependence measurements for the lipid on the nanoantennas (L1, L2 and L3) and before subtraction of the nanonatennas signals itself (Figure S4A–C), the profile of the spectra is quite similar, and a small decrease in the intensity with increasing temperature is seen. The enhancement itself is reduced at higher temperatures (see Figure S4A–C) without a change in the shape of the nanoantenna signal or its position. In order to highlight the lipid absorptions, difference spectra were obtained by subtracting the absorbance spectra of the nanoantennas at increased temperature values (25 to 75 °C) from the spectra of the lipid on the nanoantennas at the corresponding temperature (25 to 75 °C). Changes in frequency, width, and intensity of the ν_s,as_(CH_2_) bands of the lipid (slight shift of 2 cm^−1^ to higher frequencies and a slight decrease of the intensity) were observed within the same temperature range (Figure 4B). The increase in the frequency of ν_s_(CH_2_) and ν_as_(CH_2_) bands to 2863 and 2927 cm^−1^ at high temperature (75 °C) points to an increase in conformational disorder. A small decrease in the frequency of the ester ν(C=O) band (about 2 cm^−1^) (Figure 4E) in the whole temperature range accompanied by a decrease in the frequency of the ν_as_(PO_2_^−^) band (about 5 cm^−1^ at 75 °C) (Figure 4H) were seen for the lipid on nanoantennas.

Finally, the thiolipid sample immobilized on gold nanoparticles surface is characterized by a slight shift to lower frequencies of the ν_s,as_(CH_2_) bands of about 2 cm^−1^ (Figure 4C), indicating a less ordered hydrocarbon chains. The ν(C=O) band centered at 1727 cm^−1^ in the spectrum of the DPPTE on AuNPs at 25 °C (Figure 4F, black line) split into two bands located at 1712 and 1747 cm^−1^ (Figure 4F). This type of signal split was previously observed for the DPPC lipid [40] and explained by the change of the intermolecular interactions between the vibrations of the carbonyl groups at higher temperature. The bands of the ν_as_(PO_2_^−^) mode at 1226 and 1261 cm^−1^ (Figure 4I, black line) do not shift, however a significant increase in the bandwidth and a decrease of their intensities were observed at higher temperature (Figure 4I). Again, this behavior is based on the changes of the intermolecular interactions at higher temperature.

### 2.5. Temperature Phase Transition of DPPTE Bilayers on Different Gold Substrates

**Figure 5:**
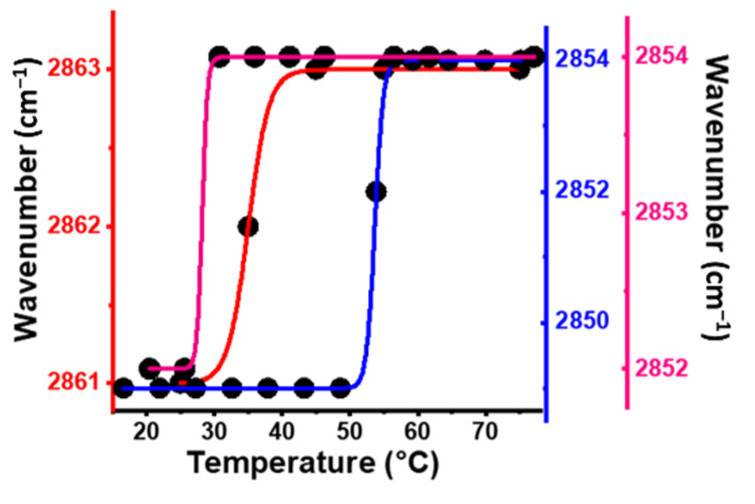
Comparison of the temperature dependence of the frequency of the ν_s_(CH_2_) band in the infrared spectra of dried DPPTE lipid on the gold layer (blue line), on the L1 gold nanoantennas (red line) and on the gold nanoparticles (magenta line).

In Figure 5, the phase transition temperature of the thiophospholipid DPPTE bilayers dried on gold nanoantennas, gold layer and AuNPs substrates was determined by plotting, the frequency of the CH_2_ symmetric stretching vibration in function of the temperature. The ν_s_(CH_2_) vibrational mode is widely used as a marker band to monitor and characterize the lipid chain-melting phase transition accurately [36,41,42].

The temperature profile of the thiolated lipid on gold layer (Figure 5, blue line) showed an abrupt increase of the slope of the curve at about 55 °C. At this temperature, the dried lipid sample on gold layer undergoes a phase transition, with spectroscopic changes specific to the transition from a liquid-crystalline phase (lamellar) to an inverted hexagonal phase (non-lamellar) [43]. The shift in the ν_s_(CH_2_) band of the thiolipid adsorbed on gold nanoantenna (Figure 5, red line) from 2861 to 2863 cm^−1^ point towards a gel to liquid-crystalline phase transition at about 35 °C. The temperature dependence of the ν_s_(CH_2_) vibrational mode measured for the lipid sample on the gold nanoparticles surface (Figure 5, magenta line) display a phase transition at temperature of about 28 °C, and although the observed spectroscopic changes in Figure 4C,F,I were small, we suggest that a lamellar-hexagonal phase transition is taking place [43].

A direct comparison of the temperature dependence spectra of thiolated lipid immobilized on gold nanoantenna and AuNPs substrate (Figure 4B,C,E,F,H,I) reveals different spectroscopic changes of the vibrational modes studied for three characteristic functional groups. This would point towards a different phase structure and organization of the phospholipids induced by temperature on both surfaces; however, the value of the phase transition temperature is similar for these different surfaces (Figure 5 red and magenta lines). Importantly, this confirms that at least partially the different spectral signatures observed for the same phospholipid on gold surfaces are not defined by the direct organization of the lipid molecules alone, but also by the resonance effects induced by the nanoantennas and the AuNPs.

In previous study on the phase behavior of DPPTE lipid by means of infrared reflection absorption spectroscopy (IR-RAS), the lipid layer supported on a gold surface after 18 h incubation at room temperature in ethanol solution demonstrated the presence of domain structures of two phases, the liquid-expanded and liquid-condensed phases [21]. The phase transition temperature determined for the DPPTE lipid using fluorescence anisotropy and liposomes made from the phospholipid in phosphate buffer solution was about 41.1 ± 0.4 °C [21], and 45 °C for crystalline DPPTE measured by calorimetry [44]. It is noted, that the experiments reported have been made at different experimental conditions, especially concerning the lipid layer creation.

## 3. Materials and Methods

### 3.1. FDTD Simulations

**Figure 6:**
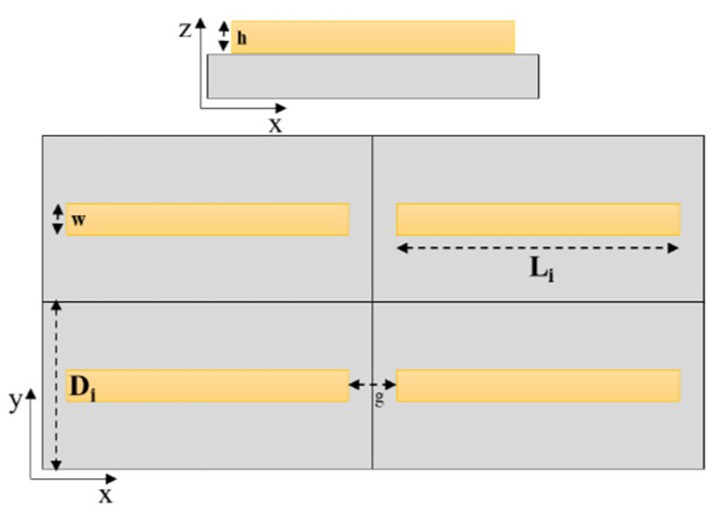
Schematic representation of the gold nanoantennas showing the geometric parameters used in the FDTD simulation.

The spectral and near-field characteristics of the nanoantenna arrays were calculated using FDTD Lumerical based on the finite element method. A plane wave source was used for plasmonic excitation of the array unit cell of length (L1 = 630 nm, L2 = 990 nm, and L3 = 1350 nm) while the period in the y-direction was (D1 = 700 nm, D2 = 1400 nm, D3 = 2000 nm, respectively), and the spacing between the nanoantennas g in the x-direction was taken to be 200 nm (Figure 6). The thickness of the nanoantennas is 100 nm while the width of the antennas is 150 nm (parameters h and w, respectively, Figure 6).

The geometrical parameters and the periods are optimized in a way to have the maximum enhancement from the nanoantennas and at the same time suitable for the fabrication procedure. It is known that the plasmonic resonance may be damped due to radiative losses in the infrared region. The control of these kind of losses could be done through coupling the plasmonic modes to lattice modes in the periodic structure and hence higher enhancement [45]. The incident light was linearly polarized with the incident electric field (EF) directed along the antenna length. The dielectric values of gold and water were from Kischkat [46] whereas a constant refractive index of 3.4 was used for the Si substrate. A thin (8 nm) Titanium (Ti) adhesion layer, present in the fabricated samples, was omitted from the simulations.

### 3.2. Nanoantenna Fabrication

Nanoantennas were nanofabricated on a Si substrate by electron beam lithography and a lift off process. Poly(methyl mathacrylate) (PMMA) was used as the pattern-defining electron beam-resist, and a lower molecular weight sublayer is used to help the lift-off process. Nanoantennas with a lateral size of 100 μm × 100 μm were exposed with a 20 keV electron beam and developed in MiBK/IPA 1:3 solution. Gold nanoantennas were formed by electron beam evaporation of an 8 nm thick adhesion layer and a 72 nm thick Au layer, followed by a lift-off process carried out in acetone, and then rinsed in ethanol and dried under nitrogen.

### 3.3. Preparation of Gold Substrates

#### 3.3.1. Gold Layer Deposition

A 7 nm layer of Au was deposited on Si substrate by e-beam-assisted evaporation on a Plassys ME300 device. After pumping to the base-pressure of 2.8 × 10^–7^ mbar, evaporation was proceeded at the working pressure of 1.2 × 10^−5^ mbar at a rate of ~0.05 nm/s. The thickness was monitored with a quartz crystal sensor.

#### 3.3.2. Deposition of Gold Nanostructures

A three-dimensional network of gold nanoparticles (AuNPs) was formed on the surface of Si substrate as previously described [47]. First the silicon substrate was polished with fine grade 0.3 μm alumina, rinsed with distilled water, acetone, and again water. The Si substrate was immersed in a solution containing ammonium fluoride (NH_4_F) (wt/vol) 40% for 1 min to remove the Si oxide layer and terminate it with hydrogen. Then, the surface was rinsed with water and dried again. The Si substrate and the plating solution were heated together at 65 °C for 10 min. The solution is a 1:1:1 mix (vol/vol/vol) of 15 mM NaAuCl_4_ + 150 mM Na_2_SO_3_ + 50 mM Na_2_S_2_O_3_ + 50 mM NH_4_ Cl, and 2% HF (wt/vol: 1 mL). The Si substrate was covered with the plating solution for 40 s, and deposition was stopped by washing the Si surface with water, followed by drying the surface with a stream of argon.

### 3.4. Formation of Supported DPPTE Lipid Bilayers on Gold Substrates

1,2-Dipalmitoyl-sn-glycero-3-phosphothioethanol (sodium salt; DPPTE) powder (~99% purity) was purchased from Avanti Polar Lipids. The gold nanoparticles network, the ultrathin gold layer fabricated onto Si wafers and gold nanoantennas substrates were used to investigate the DPPTE layers. 1 mM solution of DPPTE was prepared by dissolving the powder in chloroform and the gold substrates (AuNPs, gold layer and Au nanoantennas, separately) were incubated into the lipid solution at room temperature (25 °C) for 18 h. After 18 h of immersion, the gold substrates were carefully rinsed with chloroform (~99.9% grade) to remove physically adsorbed phospholipids from the surface, and then dried under a gentle argon flow. The immobilization procedure used here was similar to that one reported by Raffaella Lettieri et al. [21].

### 3.5. Infrared Microscopy and Temperature Dependence of the IR Spectra

The temperature dependent IR measurements of the DPPTE lipid adsorbed on the AuNPs, gold layer and nanoantennas substrates were performed in the mid-infrared (MIR) spectral range in the transmission mode. The starting temperature measurement was 20 °C and then increased until 75 °C by a step of 10 °C. The IR spectra were recorded after 10 min stability time for each temperature measurement.

IR measurements on nanoantennas were carried out using a Fourier transform IR spectrometer, Vertex 80v from Bruker optics (Karlsruhe, Germany) coupled to an IR microscope (Bruker IRScope II) equipped with a reflective reverse Cassegrain objective (NA = 0.4, 15×magnification) and a liquid nitrogen cooled mercury cadmium telluride detector. The interferometer was kept under vacuum while the microscope, including the stage, was purged with dry nitrogen. The incident light polarization was applied parallel to the axis of the nanoantennas. The knife edge apertures of the microscope were aligned to the area of the nanoantennas which is 100 × 100 μm. A bare silicon substrate was used as a background for reference measurements. Typically, three spectra of the lipid on nanoantennas (2452 co-added scans) were recorded at each temperature with a resolution of 4 cm^−1^ and velocity of 20 kHz in the spectral ranges from 4000 to 750 cm^−1^. The stage under the microscope was connected to a Thermo Scientific SC150-A28 Arctic Series refrigerated heated bath circulator, which is responsible for the increase in temperature. The lipid spectra shown here were obtained on the nanoantennas oriented in a parallel polarization (Figure S5), that corresponds to plasmonic resonance on the vibrational signal.

IR spectra of the thiolipid on AuNPs and gold film substrates were recorded with a Vertex 70 instrument from Bruker (Karlsruhe, Germany). The Au substrates coated by the DPPTE thiolipid were placed in a metallic sample holder mounted in the sample comparment of the spectrometer. The temperature close to the sample was regulated from 20 to 75 °C with a cooling system (Huber, Offenburg, Germany) connected to the sample holder. The spectrometer was purged with dry air to avoid contributions from humidity in the spectra. The IR spectra of the lipid on the gold substrates were recorded from 4000 to 600 cm^−1^ using a deuterated triglycine sulfate (dTGS) detector and a scan velocity of 10 kHz. Three non-polarized spectra of the lipid on gold layer and AuNPs were measured at each temperature with a resolution of 4 cm^−1^ (256 scans).

### 3.6. Data Analysis of the IR Spectra

The temperature dependence of the IR absorbance spectra of the lipid bilayers on the gold substrates (nanoantennas, AuNPs and Au layer) allows us to observe the main vibrational bands of the lipid. The IR spectra of the lipid were smoothed with 13 points and a straight baseline correction was applied. In order to extract the vibrational bands of the lipid from the overall absorption of the nanoantennas, the L1, L2 and L3 Au nanoantennas absorptions without lipids were interactively subtracted from the temperature dependent data (Figure S4).

### 3.7. Scanning Electron Microscopy (SEM)

SEM image of the 900 nm long nanoantenna sample have been obtained with a Zeiss Supra 40 from Zeiss Research Microscopy (Jena, Germany) (Figure 1F).

### 3.8. AFM Imaging

#### 3.8.1. AFM Imaging of Gold Nanoantenna after the Heat Treatment

AFM microscopy was used to characterize the roughness and the surface morphology of the gold nanoantenna substrate before lipid immobilization. AFM imaging was performed with a Bruker Dimension Edge in the tapping mode. In order to exclude an effect of the temperature on the nanonatennas structure, control experiments with a gold nanoantenna with a length of 3500 nm were performed by AFM at both temperatures, 25 and 75 °C (Figure S1A,B). The nanoantenna sample shown was heated at 75 °C during 44 h on a hotplate.

#### 3.8.2. AFM Imaging of the DPPTE Lipid Bilayers on Gold Substrates

The Atomic Force Microscopy Multimode Nanoscope IV from Bruker (Palaiseau, France) was used to characterize the nanostructured gold surfaces (plain gold layer and nanoantennas substrates, both, with and without lipid bilayers). The plain gold layer and Au nanoantennas substrates were prepared using the immobilization procedure described above. The lipid immobilization on the substrates was observed before chloroform rinsing for control purposes (data not shown) and after chloroform rinsing.

To probe the samples topography, a Peak Force Tapping mode (ScanAsyst) in dry state was selected using silicon nitride tips (SCANASYST-AIR from Bruker, having a spring constant “k” of around 0.4 Nm^−1^). The images were obtained preferably along the fast scan axis at a scan rate of 1 Hz with a resolution of 512 × 512 pixels. The data analysis was performed using NanoScope Analysis Software (version 1.7) from Bruker (Palaiseau, France).

## 4. Conclusions

In summary, we have used the enhancement of plasmonic infrared nanoantennas and the chemical specificity of infrared spectroscopy to investigate the organization of DPPTE tethering lipid model membrane. The combination of SEIRAS with temperature dependence analysis allows to monitor in situ the conformation and dynamics of the functional groups of the thiophospholipid on the nanostructured surface and determine its phase transition temperature.

The suitability of in-plane nanoantenna arrays has been validated on thiolated lipid membrane obtaining self-assembled bilayers with 20 nm average height. The phase transition temperature of the DPPTE lipid was determined to be about 35 °C on gold nanoantenna. We showed that the multi-resonant mid-IR nanoantennas provide relative intensity enhancements up to six and thirty orders of magnitude for the methylene bands of the lipid, when directly compared to the data obtained on plain gold layer and AuNPs surface, respectively. A modification of the profile of the vibrational signals of CH_2_, C=O, and PO_2_^−^, functional groups of the lipid was observed on the nanoantennas. This observation seems to be linked to the plasmonic resonance effect of the respective nanoantennas, as experimentally and theoretically shown [3,9]. The understanding of this phenomenon is crucial for the future application of nanoantennas as a tool in analytical sciences.

## Data Availability

Not applicable.

## Supplementary Materials

The following supporting information can be downloaded. Figure S1: AFM images of gold nanoantenna with length of 3500 nm before (at 25 °C) (A) and after (B) the heat treatment at 75 °C during 44 h, Figure S2: AFM transversal cross-section profiles showing the width of Au nanoantennas (A) before and (B) after DPPTE lipid immobilization followed by the chloroform rinsing, Figure S3: AFM longitudinal cross-section profiles (A,B) on the top and (C,D) between of Au nanoantennas; before (A,C) and after DPPTE lipid immobilization followed by the chloroform rinsing (B,D), Figure S4: Absorbance FTIR spectra of the lipid immobilized on the Au nanoantenna in function of the temperature recorded in the three different spectral regions of (A) CH_2_, (B) CO and (C) PO_2_^−^ before subtraction with the spectrum of the nanoantenna measured at 25 °C, Figure S5: Absorbance FTIR spectra of the lipid immobilized on the nanoantennas in parallel and perpendicular polarization: (A) CH_2_, (B) CO and (C) asymmetric PO_2_^−^ stretching bands of the lipid at 25 °C.

## Sample Availability

Samples of the compounds are not available from the authors.
