# Supplementary material for: Plasmonic Resonant Nanoantennas Induce Changes in the Shape and the Intensity of Infrared Spectra of Phospholipids
Source: Molecules. 2021 Dec 23;27(1):62. doi: 10.3390/molecules27010062 (PMC8746598; doi:10.3390/molecules27010062)
Supplement: Supplementary file 1 [file molecules-27-00062-s001.zip › molecules-1517080-supplementary.pdf]

Supporting Information

Plasmonic resonant nanoantennas induce changes in the shape and intensity on infrared spectra of phospholipids

Fatima Omeis[1,2+], Zahia Boubegtiten-Fezoua[1+], Ana Filipa Santos Seica[1], Romain Bernard[3], Muhammad Haseeb Iqbal[4], Nicolas Javahiraly[1], Robrecht M. A. Vergauwe[5], Hicham Majjad[3], Fouzia Boulmedais[4], David Moss[6], Petra Hellwig[1,2*]

1. Laboratoire de Bioélectrochimie et Spectroscopie, UMR 7140, CMC, Université de Strasbourg CNRS, 4 Rue Blaise Pascal 67081, Strasbourg, France

2. University of Strasbourg Institute for advanced Studies (USIAS), 4 Rue Blaise Pascal, 67081 Strasbourg, France

3. Institut de Physique et Chimie des Matériaux de Strasbourg, Université de Strasbourg, CNRS UMR 7504, 23 rue du Loess, BP 43, F-67034 Strasbourg Cedex 2, France

4. University of Strasbourg, CNRS, Institut Charles Sadron, UPR 22, 67034 Strasbourg, France

5. NanoScience Center, Survontie 9 C, 40500 Jyväskylä, Finland

6. Institute for Beam Physics and Technology, Karlsruhe Institute of Technology, Hermann-von-Helmholtz-Platz 1, D-76344 Eggenstein-Leopoldshafen, Germany

*corresponding author: hellwig@unistra.fr

+ both authors contributed equally to the study

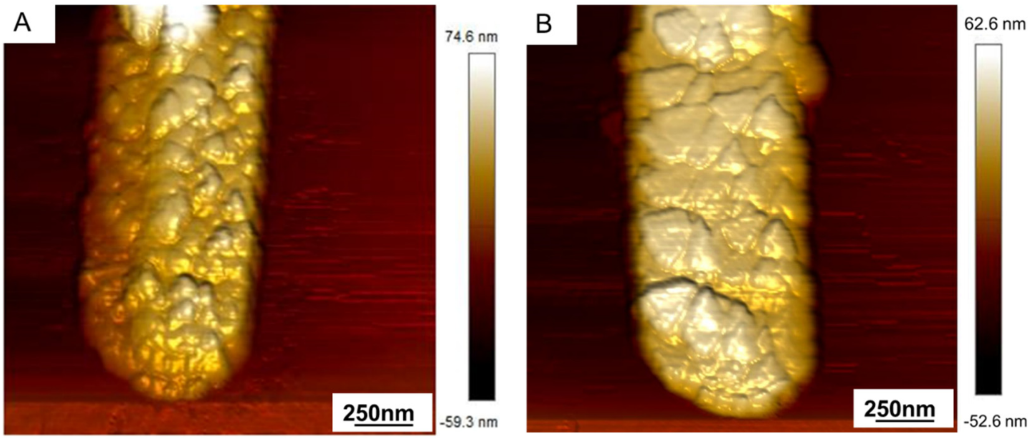

Figure S1: AFM images of gold nanoantenna with length of 3500 nm before (at 25°C) (A) and after (B) the heat treatment at 75°C during 44h.

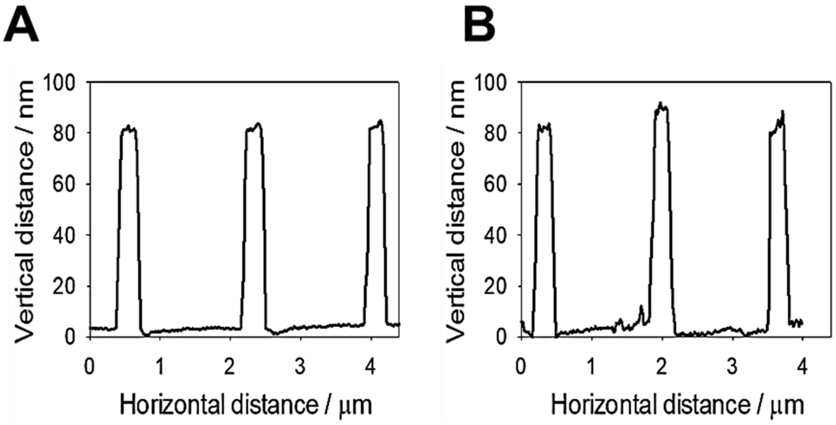

Figure S2: AFM transversal cross-section profiles showing the width of Au nanoantennas (A) before and (B) after DPPTE lipid immobilization followed by the chloroform rinsing.

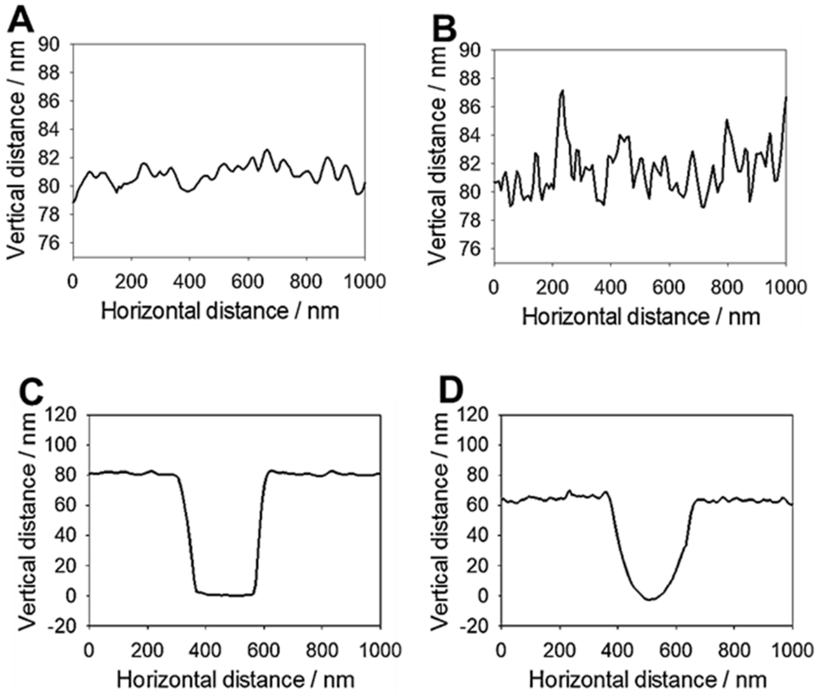

Figure S3: AFM longitudinal cross-section profiles (A, B) on the top and (C, D) between of Au nanoantennas; before (A, C) and after DPPTE lipid immobilization followed by the chloroform rinsing (B, D).

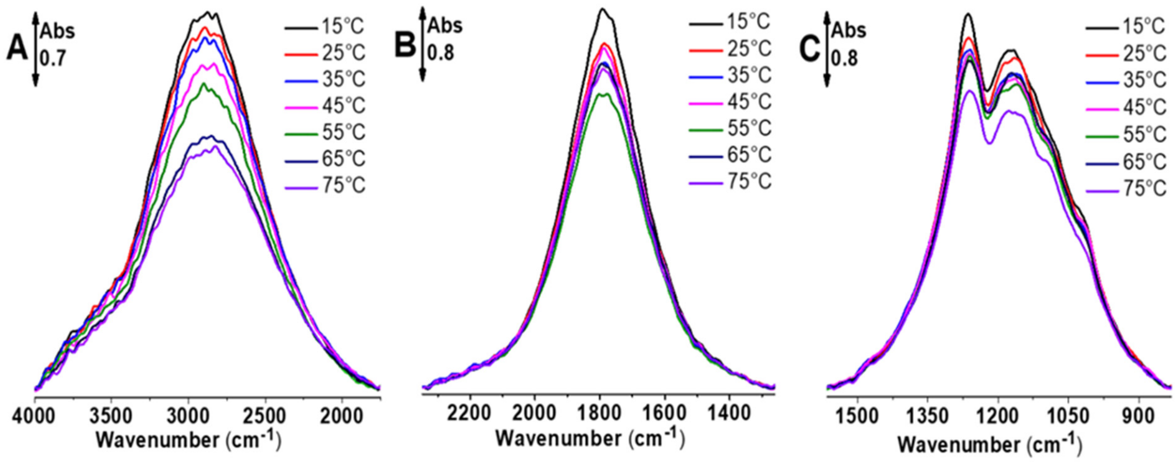

Figure S4: Absorbance FTIR spectra of the lipid immobilized on the Au nanoantenna in function of the temperature recorded in the three different spectral regions of A) CH_2, B) CO and C) PO_2^- before subtraction with the spectrum of the nanoantenna measured at 25 °C.

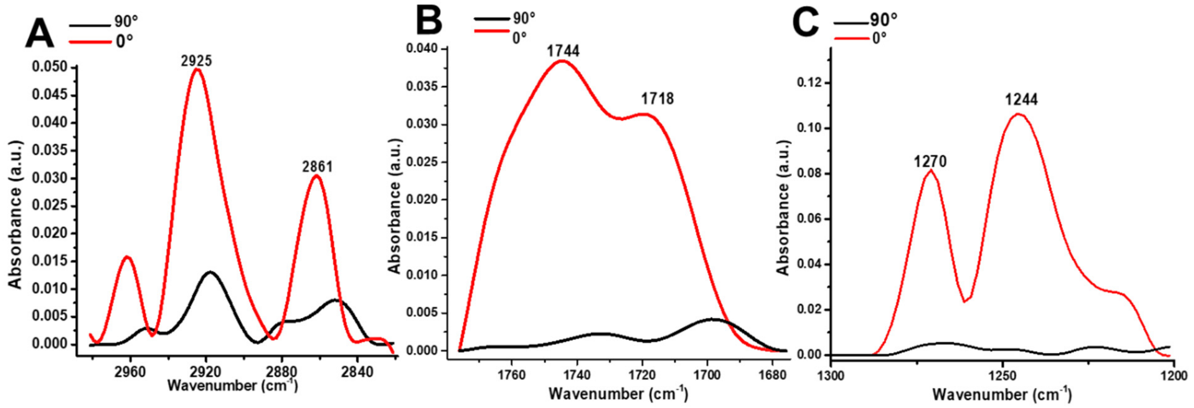

Figure S5: Absorbance FTIR spectra of the lipid immobilized on the nanoantennas in parallel and perpendicular polarization: A) CH_2, B) CO and C) asymmetric PO_2^- stretching bands of the lipid at 25°C.

.
